# Differential expression of miR-17∼92 identifies BCL2 as a therapeutic target in BCR-ABL-positive B-lineage acute lymphoblastic leukemia

**DOI:** 10.1038/leu.2013.361

**Published:** 2013-12-20

**Authors:** M Scherr, A Elder, K Battmer, D Barzan, S Bomken, M Ricke-Hoch, A Schröder, L Venturini, H J Blair, J Vormoor, O Ottmann, A Ganser, A Pich, D Hilfiker-Kleiner, O Heidenreich, M Eder

**Affiliations:** 1 Department of Hematology, Hemostasis, Oncology and Stem Cell Transplantation, Hannover Medical School, Hannover, Germany; 2 Newcastle Cancer Centre at the Northern Institute for Cancer Research, Newcastle University, Newcastle upon Tyne, UK; 3 Department of Paediatric and Adolescent Haematology and Oncology, Great North Children's Hospital, Newcastle upon Tyne, UK; 4 Department of Cardiology and Angiology, Hannover Medical School, Hannover, Germany; 5 Department of Toxicology, Hannover Medical School, Hannover, Germany; 6 Department of Hematology/Oncology and Infectious Diseases, J.W. Goethe-University Hospital Frankfurt, Frankfurt, Germany

**Keywords:** BCR-ABL, BCL2, acute lymphoblastic leukaemia, miRNA-17–92

## Abstract

Despite advances in allogeneic stem cell transplantation, BCR-ABL-positive acute lymphoblastic leukaemia (ALL) remains a high-risk disease, necessitating the development of novel treatment strategies. As the known oncomir, miR-17∼92, is regulated by BCR-ABL fusion in chronic myeloid leukaemia, we investigated its role in BCR-ABL translocated ALL. miR-17∼92-encoded miRNAs were significantly less abundant in BCR-ABL-positive as compared to -negative ALL-cells and overexpression of miR-17∼19b triggered apoptosis in a BCR-ABL-dependent manner. Stable isotope labelling of amino acids in culture (SILAC) followed by liquid chromatography and mass spectroscopy (LC-MS) identified several apoptosis-related proteins including Bcl2 as potential targets of miR-17∼19b. We validated Bcl2 as a direct target of this miRNA cluster in mice and humans, and, similar to miR-17∼19b overexpression, Bcl2-specific RNAi strongly induced apoptosis in BCR-ABL-positive cells. Furthermore, BCR-ABL-positive human ALL cell lines were more sensitive to pharmacological BCL2 inhibition than negative ones. Finally, in a xenograft model using patient-derived leukaemic blasts, real-time, *in vivo* imaging confirmed pharmacological inhibition of BCL2 as a new therapeutic strategy in BCR-ABL-positive ALL. These data demonstrate the role of miR-17∼92 in regulation of apoptosis, and identify BCL2 as a therapeutic target of particular relevance in BCR-ABL-positive ALL.

## Introduction

Acute lymphoblastic leukaemia (ALL) is a heterogeneous disease with multiple, prognostically relevant genetic aberrations. In adults, 30–40% of patients with precursor-B ALL express the BCR-ABL oncogene as the result of the Philadelphia-translocation *t*(9;22)(q34;q11) defining a very high-risk profile.^1, 2^ The BCR-ABL oncoprotein is a constitutively active tyrosine kinase involved in hematopoietic cell transformation. Inhibition of its enzymatic activity by specific tyrosine kinase inhibitors has substantially improved and fundamentally changed the treatment of chronic myeloid leukaemia and enhanced cure rates in BCR-ABL-positive childhood ALL.^3, 4^ However, treatment of adult BCR-ABL-positive ALL remains challenging. The historically poor outcome of Ph+ ALL patients has been substantially improved by combining tyrosine kinase inhibitors with induction and post-remission chemotherapy, resulting in higher remission rates and therefore a greater proportion of patients undergoing allogeneic hematopoietic stem cell transplantation (SCT).^5, 6, 7^ At present, myeloablative conditioning followed by allogeneic SCT remains the only established curative therapy, with several prospective trials showing an overall survival of 30–65%.^8, 9, 10^ Despite these advances, high transplant-associated mortality and relapse remain considerable obstacles, and the majority of elderly patients are not considered suitable candidates for allogeneic SCT, resulting in a still dismal long-term outcome.^6^ This has not been altered dramatically by the recent availability of more potent second generation tyrosine kinase inhibitors,^11^ and most likely involves mechanisms of resistance distinct from BCR-ABL tyrosine kinase domain mutations.^12^ Therefore, it is important to develop new targeted agents alongside tyrosine kinase inhibitors to improve survival and reduce morbidity in those patients currently able to undergo allogeneic SCT as well as to offer improved pharmacotherapy for patients not suitable for SCT.

miRNAs are a class of small non-coding RNAs involved in posttranscriptional control of gene expression. Upon processing, miRNAs are incorporated in an effector complex RNA induced silencing complex (RISC) which is recruited to at least partially complementary sites in target-gene mRNAs. Individual miRNAs can bind to multiple mRNAs with differential effects on gene expression of multiple targets. We hypothesised that miRNAs may be used to identify potential leukaemia-relevant therapeutic targets if they are differentially expressed in tumour cells and if they are linked to disease-relevant phenotypes.

The polycistronic microRNA cluster miR-17∼92 encodes miR-17, miR-18a, miR-19a, miR-20a, miR-19b-1 and miR-92-1.^13^ Notably, miR-17∼92-deficient mice suffer significant developmental cardiac defects and lung hypoplasia though interrogation of haematopoiesis identified isolated defects in B-lineage development.^14^ Moreover, we observed a high expression of miR-17∼92 in adult heart and in postnatal cardiomyocytes. miR-17∼92 has also been strongly implicated in both solid and haematopoietic malignancies.^15, 16^ Of these, the first and now best-studied group is the mature B-lymphoid malignancies.^17^

Conditional knockout of the cluster-revealed modulation of apoptosis as the predominant mechanism of action of miR-17∼92.^18^ In normal lymphopoiesis, loss of miR-17∼92 results in upregulation of Bim (Bcl2l11) and increased apoptosis, inhibiting the pro-B to pre-B transition.^14^ Conversely, moderate overexpression of miR-17∼92 causes a reduction in Bim and Pten expression, resulting in lymphoproliferation and autoimmune disease.^19^ Dissection of the miR-17∼92 cluster has demonstrated that miR-19 is both necessary and sufficient to abrogate apoptosis, at least in Myc-mediated lymphomagenesis most likely by repression of PTEN and BIM.^18, 20^

Based on our previous work in chronic myeloid leukaemia,^21^ we first analysed miR-17∼92 expression in ALL and observed a significantly lower expression in ALL as compared to normal CD34+ cells with further reduction in BCR-ABL-positive as compared to -negative ALL cells. We next over-expressed the miR-17∼92 derivative miR-17∼19b in an inducible model of BCR-ABL-positive ALL, thereby identifying impaired apoptosis as a key determinant of reduced miR-17∼92 function in this disease setting. Quantitative proteome-wide expression analysis and alignment of miR-17∼19b seed regions identified multiple apoptosis regulators as being downregulated by miR-17∼19b-overexpression. From these, *BCL2* was validated as a direct target of the miR-17 and miR-18a, and *BCL2* knockdown resulted in strong induction of apoptosis in BCR-ABL-positive, but not BCR-ABL-negative ALL cells. Accordingly, BCR-ABL-positive cells also demonstrated a selective sensitivity to the BCL2 inhibitor ABT-737 *in vitro*. Finally, we demonstrated the sensitivity of BCR-ABL-positive ALL to ABT-737 in a real-time *in vivo* validation assay using patient-derived primary ALL cells transduced with luciferase. This study identifies *BCL2* as a potential therapeutic target in BCR-ABL-positive ALL.

## Materials and methods

### Patient material

BM and PB samples were collected from 13 and 14 newly diagnosed BCR-ABL-positive and -negative B-lineage ALL, respectively (⩾60% blasts). BM-derived CD34+ cells from four healthy volunteers served as controls. The study was approved by the Ethics Committee of the University of Frankfurt.

Patient-derived material used for mouse transplantation was collected as part of the initial diagnostic investigation of patients. It was collected, stored and used with written informed consent according to approvals given by the local institutional review boards and the Declaration of Helsinki. Samples were retrieved from Newcastle Haematological BioBank under the generic BioBank approval given by the Newcastle & North Tyneside Ethics Committee (REC reference number: 07/H0906/109).

### SILAC, LC-MS and data processing

TonB cells were cultured with either isotopically labelled Lysine (^13^C_6_-^15^N_2_-Lys) and Arginine (^13^C_6_-^15^N_4_-Arg) (heavy state), Lysine (^2^H_4_-Lys) and Arginine (^13^C_6_-Arg) (medium state) or natural Lysine and Arginine (light state). Three biological replicates were prepared with all three labelling states, light, medium and heavy, included as described recently.^22^ Cell lysates were separated by SDS-PAGE followed by gel slicing, extraction and trypsin digestion. Peptide samples were separated and fragmented with a nano-flow ultra-high pressure liquid chromatography system (RSLC, Thermo Scientific, Waltham, MA, USA) coupled online to a Nano Spray Flex Ion Source II (Thermo Scientific) of an LTQ-Orbitrap Velos mass spectrometer. Fragment ion mass spectra were recorded in the LTQ part of the mass spectrometer at a normal scan rate and stored as centroid *m/z* value and intensity pairs. Raw data were processed with the MaxQuant proteomics software (MaxQuant, Martinsried, Germany) suite version 1.1.1.36 for identification and quantification of proteins as described. Peptides and proteins were identified with the implemented Andromeda search engine version 1.1.0.36 and the human entries of the IPI protein data base (v. 3.73).

### Immunoprecipitation of human argonaute 2 complexes using the RIP-ChIP kit

Lentiviral supernatants expressing miR-17∼19b and control vector SIEW were used to transduce ∼1 × 10^6^ 293 cells with an MOI of ∼2. microRNA:mRNA immunoprecipitation was performed using the Magna RIP RNA-Binding Immunoprecipitation kit (Millipore, Billerica, MA, USA) following the manufacturer's protocol. A total of 5 × 10^6^ cells were taken for each replicate and washed in phosphate buffered saline, prior to lysis in 100 μl complete RIP-lysis buffer and overnight incubation with magnetic beads conjugated with an anti-AGO2/eIF2C2 antibody (Abcam, Cambridge, UK) or control normal mouse IgG (Millipore) at 10 °C with rotation. Coimmunoprecipitated RNA, including miRNA:mRNA complexes, was subjected to qRT-PCR and miR-qRT-PCR as described before.

### ABT-737 treatment in mouse xenotransplantation studies

Primograft material was lentivirally transduced and intrafemorally transplanted into NSG mice as described previously.^23, 24^ Mice were imaged using an IVIS Spectrum pre-clinical imaging system (Perkin Elmer). Mice were injected with either vehicle control or ABT-737 (50 mg/kg/day) for a total of 30 days (5 days on, 2 days off). Mice were kept until they exhibited clinical symptoms that necessitated humane killing. Kaplan–Meier curves were plotted and analysed using GraphPad Prism software (GraphPad, San Diego, CA, USA), with significance assessed using a Log-rank (Mantel–Cox) test. All work was conducted in accordance with the UK Home Office Project Licence PPL60/3846.

## Results

### miR-17∼92 is downregulated in BCR-ABL-positive human ALL samples

**Figure 1:**
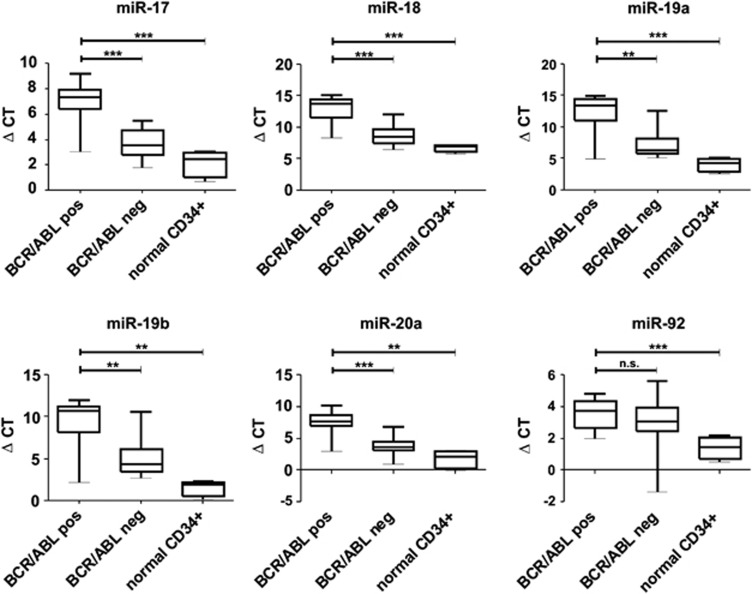
BCR-ABL+ ALL samples have a reduced expression of miR-17∼92. Differential expression of miR-17∼92-encoded miRNAs in primary BCR-ABL-positive and BCR-ABL-negative B-lineage ALL patient samples and normal CD34+ cells as determined by miR-qRT-PCR. Data are presented as ΔCT of miR-17∼92 expression in patient samples, with higher ΔCTs indicating lower expression. **P-value <0.001; ***P-value <0.0001.

We previously demonstrated an increased miR-17∼92 expression in chronic phase chronic myeloid leukaemia CD34+ cells, compared to normal CD34+ cells from healthy donors.^14^ Based on this, we analysed expression of miR-17∼92 encoded miRNAs in 14 BCR-ABL-negative and 13 BCR-ABL-positive ALL samples, as well as normal CD34+ cells, using miR-qRT-PCR. To our surprise, all individual miR-17∼92 miRNAs were less abundant in ALL as compared to normal CD34+ cells (Figure 1). Furthermore, BCR-ABL-positive ALL samples exhibited a 9- to 32-fold reduction in miRNA expression compared to BCR-ABL-negative ALL cells, with the exception of miR-92 which was therefore not further analysed.

### The miRNA polycistron is differentially expressed and induces apoptosis in a BCR-ABL-specific manner

To investigate whether differential miR-17∼92 expression is controlled by BCR-ABL, we used an inducible murine model of BCR-ABL expression. TonB cells are derived from BaF3, a B-lymphoid cell line, and have been modified to conditionally express BCR-ABL upon addition of doxycycline.^25^ In this setting, expression of BCR-ABL was associated with a 2.3–3.3-fold reduction in expression of miR-17, -18a and -19a, in agreement with our findings in primary ALL samples (Supplementary Figure 1). These results suggested that miR-17∼92 miRNAs could have previously undiscovered anti-oncogenic functions under certain circumstances.

**Figure 2:**
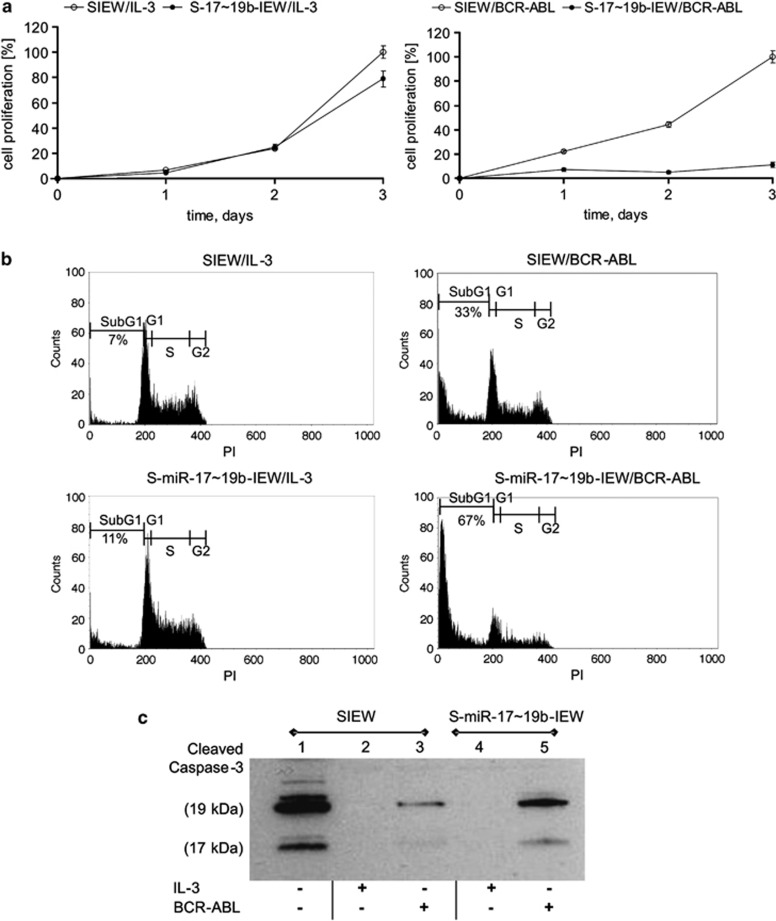
miR-17∼19b reduces proliferation and increases apoptosis in BCR-ABL+ cells (**a**) Proliferation kinetics of TonB cells expressing miR-17∼19b either in the presence of IL-3 (left) or doxycycline (right) as determined by trypan blue exclusion. Values are expressed as means±s.d. (**b**) Induction of apoptosis in TonB cells expressing miR-17∼19b (lower panels) or control vector SIEW (upper panels) in the presence of IL-3 (left) or doxycycline (right) as determined by PI staining. The percentage of apoptotic cells (subG1 phase) is shown. (**c**) Cleavage of Caspase 3 in TonB cells expressing control vector SIEW (lanes 1–3) or the miR-17∼19b polycistron (lanes 4–5) in the presence of IL-3 (lanes 2 and 4) or doxycycline (lanes 3 and 5). Whole-cell lysates were subjected to western blotting to determine cleavage of caspase 3 24 h after induction of BCR-ABL by doxycycline.

TonB cells are dependent on interleukin-3 (IL-3) for survival and growth; induction of BCR-ABL allows cytokine-independent proliferation, making them an ideal system to study miR-17∼92 function in the context of BCR-ABL. As miR-92a expression was unchanged between BCR-ABL-positive and -negative ALL cells (Figure 1), we transduced TonB cells to overexpress miR-17∼19b, a derivative of miR-17∼92 suitable for transgenic expression.^21, 26^ miRNA expression was increased between 5- and 16-fold upon transduction (miR-17 5.2-fold, miR-18a 2.1-fold, miR-19a 9-fold, miR-19b 10.6-fold, and miR-20a 15.8-fold). Proliferation of TonB cells grown in the presence of IL-3 was only slightly reduced by transgenic miR-17∼19b expression as compared to controls (SIEW) (Figure 2a, left). In contrast, BCR-ABL-mediated cell proliferation was strongly inhibited by miR-17∼19b (Figure 2a, right).

To study the impact of miR-17∼19b on cell cycle regulation and apoptosis, transgenic TonB cells were analysed for DNA content in the presence and absence of BCR-ABL. Whereas cell cycling was only marginally affected, apoptosis was markedly enhanced by overexpression of miR-17∼19b following induction of BCR-ABL. Transgenic expression of miR-17∼19b increased the subG1 fraction from 33 to 67% compared to vector controls (Figure 2b, right), whereas in BCR-ABL-negative cells it remained almost unchanged (7 and 11%, respectively, Figure 2b, left). In agreement with the DNA content analysis, increased cleavage of caspase 3 was observed in BCR-ABL expressing as compared to IL-3 supplemented cell cultures (Figure 2c, lanes 2 and 3). Overexpression of miR-17∼19b also led to a further increase in caspase 3 cleavage in BCR-ABL expressing cells (Figure 2c, lanes 3 and 5), but not in cells grown in the presence of IL-3 (Figure 2c, lanes 2 and 4). Together, these data demonstrate that miR-17∼19b decreases cell proliferation and markedly increases apoptosis in a BCR-ABL-specific manner.

### miR-17∼19b targets regulators of apoptosis

Given that miR-17∼19b overexpression was a driver of cell death in TonB cells in a BCR-ABL-specific manner, we hypothesised that identifying key targets of the cluster could provide novel therapeutic opportunities in BCR-ABL-positive ALL. miRNAs predominantly affect protein expression, so we initially used a stable isotope labelling in cell culture (SILAC)-based approach to identify miR-17∼19b- and miR-20a-regulated proteins. miR-20a overexpression showed similar, but weaker, effects to miR-17∼19b in preliminary experiments (data not shown). TonB cells metabolically labelled with heavy, medium, or light isotope lysine and arginine versions were lentivirally transduced with miR-17∼19b, miR-20a, or a control vector (SIEW). Cells were cultured with IL-3 and differential protein expression was analysed by quantitative proteomics using liquid chromatography mass spectrometry (LC-MS). Upon lentiviral transduction, miR-17∼19b miRNA expression was increased between 3- and 12-fold as compared to controls (Supplementary Figure 2A). In total, we identified 3962 proteins from TonB cell lysates and were able to determine the abundance of 3541 of these proteins under all three labelling conditions (Supplementary Table 1A). The correlation between the three labelling conditions was high, with *r*^2^ between 0.54 and 0.65 (Supplementary Figure 2B). In total, 84 proteins were regulated more than 1.7-fold by miR-17∼19b, with 31 exhibiting higher abundance and 53 exhibiting lower abundance in miR-17∼19b transgenic TonB cells. Protein abundance was only slightly affected in miR-20a cells and thus, respective proteins were not further analysed.

**Figure 3:**
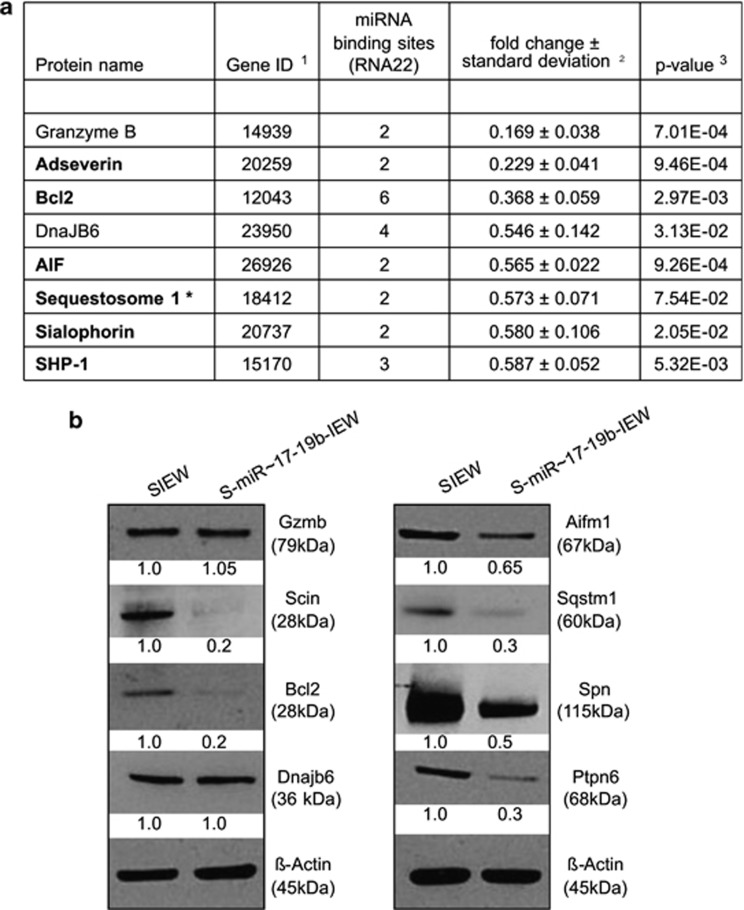
miR-17∼19b targets several apoptosis-related proteins. (**a**) Downregulated proteins involved in the regulation of apoptosis. Reduced protein expression (bold) was validated by immunoblotting as shown in (**b**). ^1^Gene ID according to NCBI (http://www.ncbi.nlm.nih.gov/); ^2^mean value of *n*=3 experiments, *n=2 experiments; ^3^*t*-test; two-tailed; paired. (**b**) Western blotting showing Granzyme B (Gzmb), Adseverin (Scin), Bcl2, DnaJB6, Aif (Aifm-1), Sequestosome-1 (Sqstm1), Sialophorin (Spn) and Shp-1 (Ptpn6) protein expression in TonB cells lentivirally transduced with control vector SIEW (left) or miR-17∼19b (right). Relative expression levels were normalised to β-actin, which served as a loading control.

We next categorised the 53 low abundance proteins into functional groups using GeneCoDis 2.0. Gene enrichment analysis revealed 269 hits in 102 different gene ontology groups (Supplementary Figure 2C). In keeping with the enriched subG1 fraction and caspase 3 cleavage pattern, ‘regulation of apoptosis' (GO: 0042981) contained eight genes (Figure 3a), and was the largest single group of all identified gene ontologies. These findings were confirmed by the KEGG pathway analysis (Supplementary Table 1B). We further validated the apoptosis-related candidate targets by western blotting. As shown in Figure 3b, protein expression of Adseverin (Scin), Bcl2, Sialophorin (Spn), Aifm-1 (Aif), Sequestosome-1 (Sqstm1) and Shp-1 (Ptpn6) was reduced in the presence of miR-17∼19b (from 0.2- to 0.65-fold), whereas protein levels of Granzyme B (Gzmb) and DnaJB6 remained unchanged.

These data point to a direct role for miR-17∼19b encoded miRNAs in the regulation of apoptosis in BCR-ABL-positive ALL. Overcoming apoptosis is key to leukemogenesis, and agents targeting cell death, notably corticosteroids, has a major role in the therapy of ALL. BCL2 is a well-established inhibitor of mitochondrial apoptotic pathways, and has emerged as a potential therapeutic target in both leukaemias and solid tumours.^27, 28^ We therefore decided to focus on the proteins involved in regulation of apoptosis and on BCL2 in particular.

### miR-17∼19b suppresses expression of Bcl2

We next analysed the presence of putative miR-17∼92-binding sites (seed matches) within the target mRNAs. As miRNAs binding is not restricted to exact reverse complement seed sequences within the 3′-untranslated region (UTR)^28, 29^ we used the miRNA target prediction programme RNA22 based on earlier reports of its low false prediction rate and ability to discover noncanonical targets.^29, 30, 31, 32^ As shown in Figure 3a, all targets analysed have at least two miRNA-binding sites for the miR-17∼19b cluster. Notably, six binding sites for miR-17∼19b miRNAs (three sites for miR-18a, two sites for miR-17 and one site for miR-20a) are located within the 5′UTR and CDS of murine *Bcl2* (Supplementary Figure 3A). In human *BCL2,* we identified 13 binding sites for miR-17∼19b miRNAs (five sites for miR-17, six sites for miR-18a and two sites for miR-20a) located within the CDS and 3′UTR (Supplementary Figure 3B).

**Figure 4:**
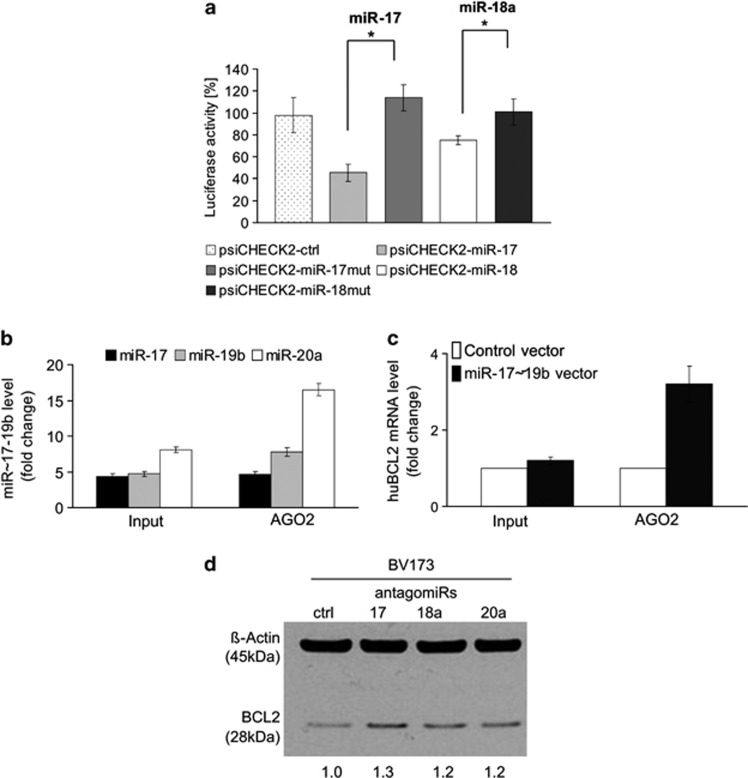
miR-17∼19b directly targets BCL2. (**a**) Bar graph of luciferase activity in stably transfected NIH3T3/S-17–19b-IEW cells co-transfected with a psiCHECK empty vector (psiCHECK-ctrl) or psiCHECK vector harbouring either the candidate wildtype or mutated (mut) miR-17 or miR-18a-binding sites in the murine Bcl2 transcript. *P-value <0.05. (**b** and **c**) RNA-binding protein immunoprecipitation (RIP) assays for AGO2 complexes in 293 cells transduced with lentiviral vectors encoding miR-17∼19b or control vector SIEW. Levels of miR-17, miR-18a and miR-20a (**b**) and BCL2 mRNA (**c**) were quantified by qRT-PCR. Graphs show expression levels in miR-17∼19b transduced cells relative to untransduced cells for both the input (left) and AGO2 immunoprecipitated fraction (right). All data are means±s.d. (*n*=3). (**d**) Western blots depicting protein levels of BCL2 after lentiviral transduction of BV173 cells with antagomirs directed against miR-17, miR-18a, miR-20a, or control miR alone.

To verify that *Bcl2* is a direct target of miR-17∼19b miRNAs, we transfected luciferase reporter constructs containing murine *Bcl2* sequences into miR-17∼19b overexpressing NIH3T3 cells. As shown in Figure 4a, miR-17∼19b significantly repressed luciferase activity for the wildtype but not for mutated miR-17 and miR-18a-binding sites in the murine *Bcl2* 5′UTR. The CDS-binding sites showed no effect on luciferase activity (data not shown). These data demonstrate that the expression of murine *Bcl2* is directly regulated by miR-17∼19b through miRNA binding within the 5′-UTR.

Having confirmed murine *Bcl2* as a target of miR-17∼19b, we next analysed specific targeting of the human *BCL2* transcript by the cluster. We used qRT-PCR to investigate the association of *BCL2* mRNA with AGO2, the catalytic component of RISC in miR-17∼19b overexpressing human 293 cells. Assessment of miR-17∼19b levels following transduction confirmed overexpression and AGO2 association of all three miRNAs (Figure 4b). Whereas levels of *BCL2* mRNA in the input fraction were unchanged following miR-17∼19b overexpression, anti-AGO2 immunoprecipitates specifically pulled down 3.2-fold more *BCL2* mRNA as compared to controls (Figure 4c). This shows that miR-17∼19b overexpression results in increased binding of *BCL2* mRNA to AGO2 demonstrating specific targeting of *BCL2* mRNA by miR-17∼19b miRNAs. We further confirmed this by a complementary approach using lentiviral overexpression of antagomirs against miR-17, miR-18 and miR-20a in the human BCR-ABL-positive BV173 cell line.^33^ All antagomirs increased *BCL2* expression by approximately 20% (Figure 4d). Together, these data demonstrate direct and functional miRNA binding of miR-17∼19b members namely miR-17/miR-20a and miR-18a to human *BCL2* mRNA. They also strongly suggested that miR-17∼19b mediated repression of *Bcl2* could contribute to the pro-apoptotic effects of the cluster in the context of BCR-ABL.

### Repression of murine and human Bcl2 expression mimics miR-17∼19b overexpression

**Figure 5:**
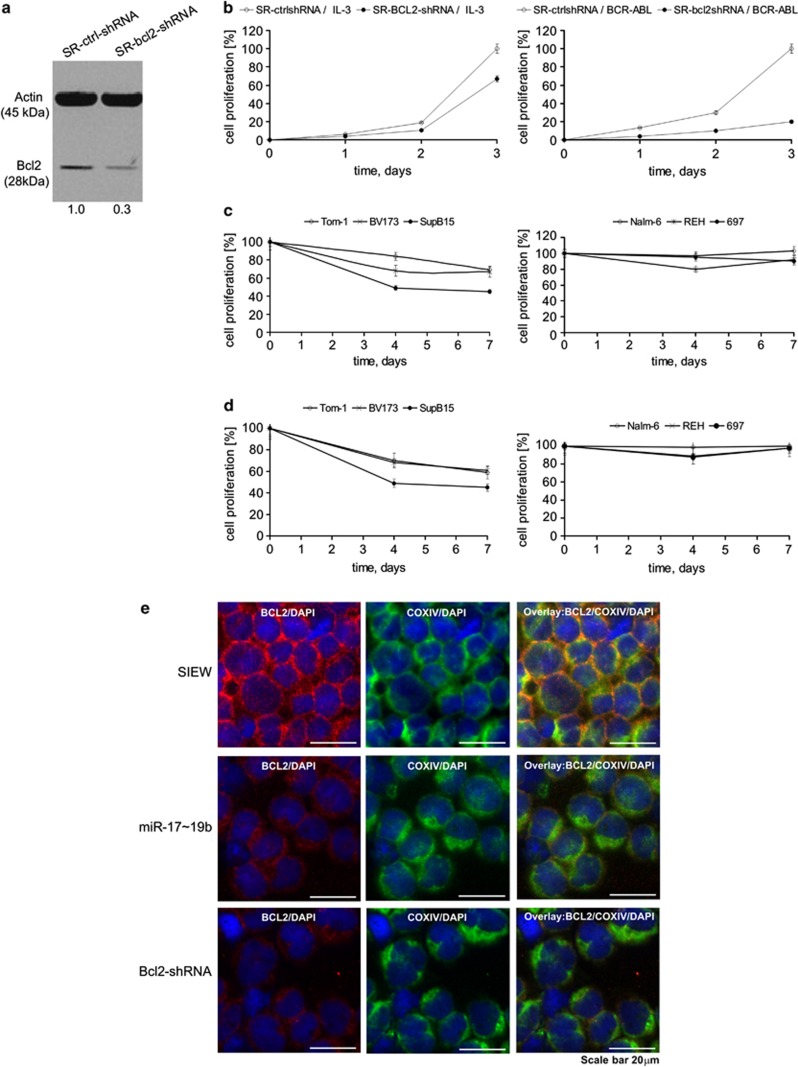
Repression of Bcl2 mimics miR-17∼19b overexpression in human and mouse. (**a**) Western blots of Bcl2 after lentiviral transduction of TonB cells with anti-Bcl2 shRNA or control shRNA. (**b**) Proliferation kinetics of TonB cells expressing anti-Bcl2 shRNA or control vector either in the presence of IL-3 (left) or doxycycline (right) as determined by trypan blue exclusion. (**c**) Proliferation kinetics of BCR-ABL-positive cell lines Tom-1, SupB15, and BV173 (left) and of BCR-ABL-negative cell lines Nalm-6, REH and 697 (right) expressing miR-17∼19b or control vector using trypan blue exclusion. (**d**) Proliferation kinetics of BCR-ABL-positive cell lines Tom-1, SupB15, or BV173 (left) and of BCR-ABL-negative cell lines Nalm-6, REH, or 697 (right) after transduction with anti-BCL2 shRNA or ctrl-shRNA using trypan blue exclusion. All values were expressed as means±s.d. (**e**) Fluorescence microscopy of SupB15 cells transduced with control vector (top), miR-17∼19b (middle), or anti-BCL2 shRNA (down) of BCL2 protein (red) or the mitochondrial marker COX IV (green). Cells were counterstained with DAPI (blue), scale bar: 20 μm.

To study the functional contribution of Bcl2 to the miR-17∼19b-induced phenotype, we lentivirally transduced TonB cells to express either anti-Bcl2- or control shRNA. Anti-Bcl2 shRNA reduced Bcl2 protein expression by ∼70% – a similar level to overexpression of miR-17∼19b (∼80%) (Figure 5a). In IL-3 supplemented cultures, expression of Bcl2 shRNA inhibited cell proliferation by about 25% compared to controls (Figure 5b, left), whereas BCR-ABL driven cell proliferation was inhibited by about 75% (Figure 5b, right) suggesting a particular requirement for Bcl2 in BCR-ABL expressing cells.

We next analysed the functional effects of miR-17∼19b overexpression in human BCR-ABL-positive ALL cell lines. As shown in Figure 5c, lentiviral transduction of miR-17∼19b into the human BCR-ABL-positive cell lines Tom-1, BV173 and SupB15 inhibited cell proliferation by 40–55% as compared to controls (Figure 5c, left). In contrast, transduction of the BCR-ABL-negative ALL cell lines REH, Nalm-6, and 697 with miR-17∼19b had no, or only minor, inhibitory effects on cell proliferation (Figure 5c, right). Expression of miR-17∼19b reduced BCL2 protein expression in BCR-ABL-positive ALL cell lines by 30–70% (Supplementary Figure 4A).

Cell lines were subsequently transduced with either anti-BCL2 or control shRNA. Anti-BCL2 shRNA substantially reduced *BCL2* mRNA expression in all cell lines (Supplementary Figure 4B). In BCR-ABL-positive Tom-1, BV173 and SupB15 cells, expression of BCL2 shRNA inhibited cell proliferation (Figure 5d, left), whereas reduction of *BCL2* mRNA expression in BCR-ABL-negative Nalm-6, REH and 697 cells did not substantially inhibit cell proliferation. (Figure 5d, right). Efficient downregulation of BCL2 protein in SupB15 cells by lentivirally mediated overexpression of both miR-17∼19b and anti-BCL2 shRNA was further confirmed by fluorescence microscopy demonstrating co-localisation of BCL2 (red) and the mitochondrial protein COX4 (green) (Figure 5e). Together, these results demonstrate a specific role for BCL2 in the proliferation of human BCR-ABL-positive ALL cells and suggest that the miR-17∼92 cluster suppresses BCL2 in BCR-ABL-positive cells.

### The BCL2 inhibitor ABT-737 specifically inhibits BCR-ABL-positive lymphoid cell lines

**Figure 6:**
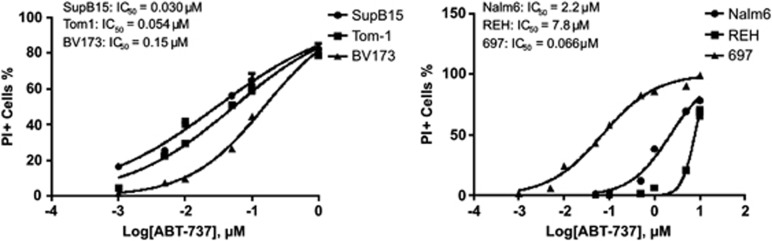
Pharmacological inhibition of BCL2 is effective in BCR-ABL+ cell lines. Cell death was assessed by uptake of propidium iodide (PI) after treatment with increasing concentrations of ABT-737 in BCR-ABL-positive cell lines (Tom-1, SupB15, BV173, left) and BCR-ABL-negative cell lines (Nalm-6, REH, 697, right) for 24 h. IC_50_ values were calculated using GraphPad Prism software.

The importance of BCL2 for the proliferation of BCR-ABL-positive cell lines suggested that BCL2 could be a highly promising novel therapeutic target. To assess the potential of pharmacological BCL2 inhibition in B-lineage ALL, BCR-ABL-positive and -negative ALL cell lines were treated with the BCL2 inhibitor ABT-737 for 24 h, prior to assessment of cell death. ABT-737 inhibited proliferation of the BCR-ABL-positive cell lines Tom-1, BV173 and SupB15 with IC_50_s of 0.054 μM, 0.15 μM and 0.030 μM, respectively (Figure 6, left). In contrast, the IC_50_ for ABT-737 was substantially higher in two out of three BCR-ABL-negative cell lines 697, Nalm-6 and REH with IC_50_s of 0.066 μM, 2.2 μM, and 7.8 μM, respectively (Figure 6, right).

### Effects of ABT-737 and Imatinib in BCR-ABL-positive ALL cells

**Figure 7:**
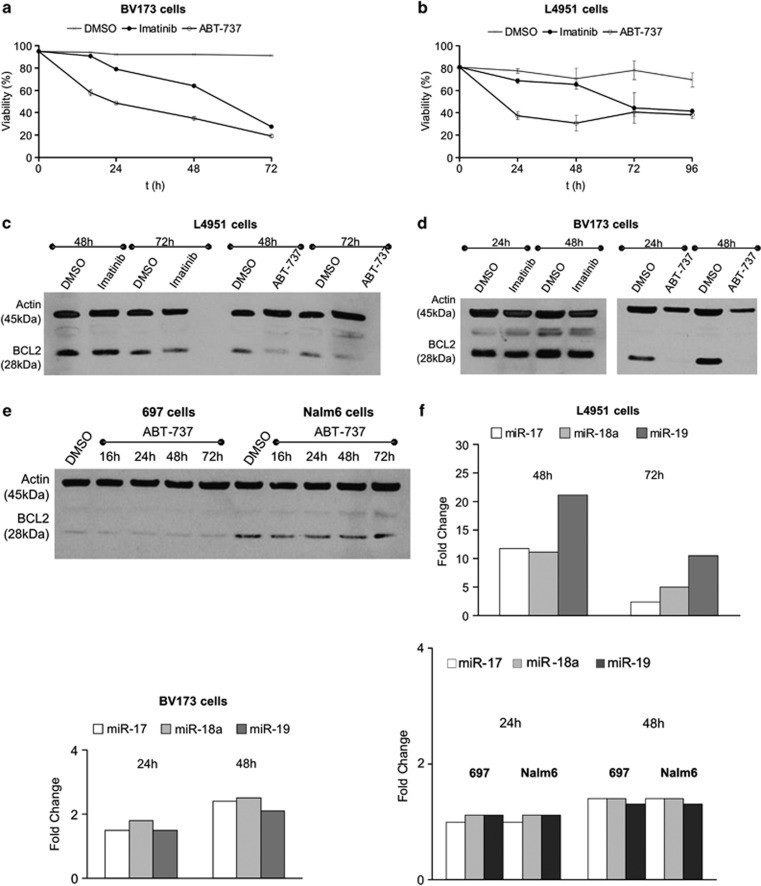
ABT-737 is more potent than Imatinib in cell lines and primary material. BV173 cells (**a**) and L4951 primograft cells (**b**) were treated with ABT-737 (0.1 μM), Imatinib (1 μM) or DMSO control and cell viability was assessed by PI uptake. (**c** and **d**) Western blots depicting protein levels of BCL2 in BCR-ABL-positive L4951 and BV173 cells after treatment with Imatinib (1 μM) or ABT-737 (1 μM) at the indicated time points. (**e**) Western blots depicting protein levels of BCL2 in BCR-ABL-negative 697 and Nalm-6 cells after treatment with ABT-737 at indicated time points. (**f**) The levels of miR-17, miR-18a and miR-19 following ABT-737 treatment were determined by miR-qRT-PCR. Graphs show fold change compared to DMSO controls.

Having demonstrated specific effects of pharmacological BCL2 inhibition in BCR-ABL-positive cells, we compared ABT-737 with imatinib in both a cell line (BV173) and a patient-derived sample. For the latter, we used primary BCR-ABL-positive precursor-B cell ALL blasts, which had been passaged through NOD/LtSz-scid IL-2Rγ null (NSG) mice (primograft, designated L4951 cells).^23^ Cells were incubated with either drug or vehicle control and the number of viable cells was determined over time. As shown in Figures 7a and b, ABT-737 induced a more rapid decline of viable cell numbers than imatinib in both BV173 and L4951 cells. Interestingly, immunoblotting revealed a rapid decrease in BCL2 protein expression following ABT-737 treatment in both primograft material and BV173 cells (Figures 7c and d). A rapid decline of BCL2 protein was also observed in SupB15 cells as early as 6 h after addition of ABT-737 (Supplementary Figure 5A). Imatinib treatment did not affect the BCL2 protein expression and this depletion was not observed in BCR-ABL-negative ALL cell lines (Figure 7e). Furthermore, after ABT-737 but not imatinib treatment, a delayed increase in expression of miR-17∼92 encoded miRNAs (starting at approximately 24 h after addition of ABT-737, Figure 7f, Supplementary Figure 5B) has been observed. These data demonstrate different mechanisms of action for ABT-737 and imatinib in BCR-ABL-positive ALL cells and suggest a role for miR-17∼92 encoded miRNAs in BCL2-mediated apoptotic pathways in these cells. They also suggest that inhibition of BCL2 by ABT-737 results in perturbations in wider signalling networks that leads to expression changes in both miR-17∼92 and BCL2 protein.

### BCL2 inhibition inhibits tumour xenograft growth *in vivo*

**Figure 8:**
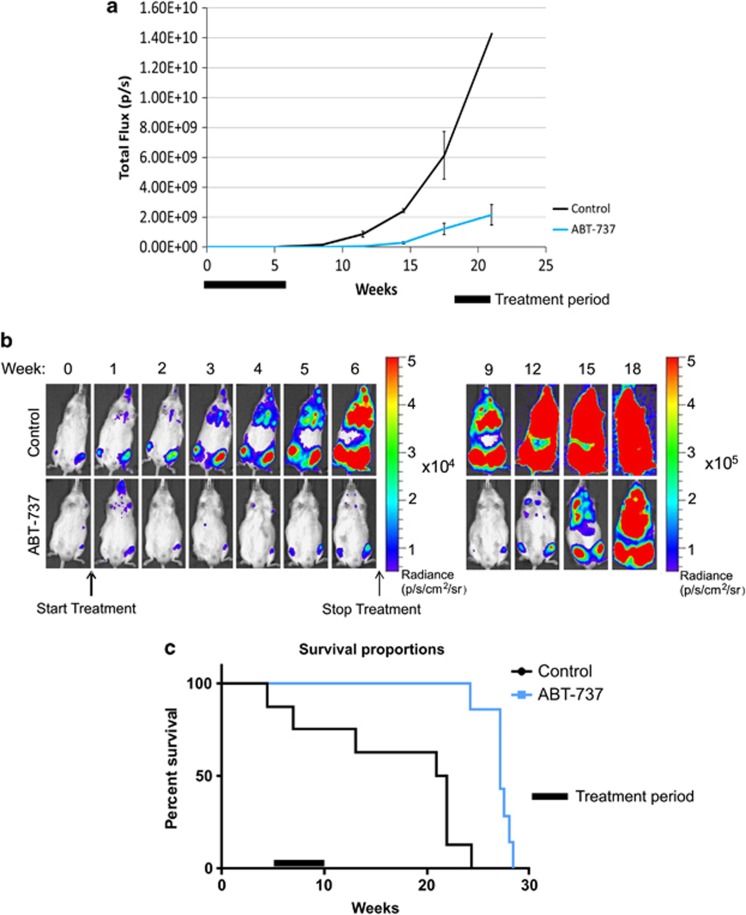
ABT-737 impairs expansion of human primary BCR-ABL+ ALL cells *in vivo*. (**a**) NSG mice were transplanted with luciferase-expressing L4951 blasts and treated with ABT-737 (50 mg/kg/day) or vehicle control for 6 weeks (black bar) following successful engraftment. Graph shows average of total flux from whole mouse at each measurement point, *n*=7 mice per group. (**b**) Images of representative mice from ABT-737 treated and control groups showing expansion of luciferase-expressing transduced blasts over time. Mice during treatment period (weeks 0–6) and after treatment (weeks 9–18) are shown on different radiance scales to avoid image saturation. Supplementary Figure 6 shows all the images on the same scale. (**c**) Kaplan–Meier survival curves for mice transplanted with L4951 blasts treated with ABT-737 (50 mg/kg/day) or vehicle control. Black bar=treatment period. Median survival 151 days (control), 190 days (ABT-737 treated), *P*=0.0004.

Finally, we examined the therapeutic potential of BCL2 inhibition by evaluating the ability of ABT-737 to inhibit growth of primary leukaemia cells *in vivo*. We developed a murine xenotransplantation assay of patient-derived primary leukaemic cells, which allows for real-time monitoring of drug therapies by bioluminescent imaging.^24^ Primograft L4951 cells were lentivirally transduced with a luciferase-expressing vector (pSLIEW, transduction efficiency ∼40%) and transplanted intrafemorally into NSG mice. Following successful engraftment determined by bioluminescent imaging, mice were injected intraperitoneally with either ABT-737 (50 mg/kg/day) or vehicle control for a total of 30 treatments. Mice treated with ABT-737 showed a substantially reduced dissemination of luciferase-expressing leukaemic blast cells over the treatment period, represented by a decrease in total photon flux emitted (Figures 8a and b, Supplementary Figure 6). In addition, ABT-737 treatment significantly lengthened the time before the mice developed clinical symptoms that necessitated humane killing, with median survival increased from 151 to 190 days (*P*=0.0004) (Figure 8c). Termination of ABT-737 treatment was followed by an increase in photon flux, demonstrating that disease control during the treatment period was being mediated by ABT-737. These results demonstrate that BCL2 inhibition is a viable treatment strategy for BCR-ABL-positive ALL *in vivo*. They also confirm the potential of our model to assess novel drug therapies on patient-derived malignant cells in an *in vivo* setting.

## Discussion

In a search for new therapeutic strategies in BCR-ABL-positive ALL, we identified targets of miRNAs with differential expression and function in these cells. As we have shown upregulation of the oncomir miR-17∼92 in chronic phase chronic myeloid leukaemia,^21^ we investigated the role of this cluster in BCR-ABL-positive ALL. To our surprise, patient samples showed significantly lower expression of mature miR-17∼92 elements in BCR-ABL-positive ALL than in either BCR-ABL-negative ALL or normal CD34+ cells. Furthermore, the TonB model of inducible BCR-ABL expression on a murine B-lymphoid precursor background demonstrated a significant reduction in mature miR-17∼92 expression following induction of BCR-ABL, confirming the specificity of this finding for BCR-ABL-positive ALL. Overexpression of the miR-17∼92 derivative miR-17∼19b resulted in reduced proliferation and notably a substantial pro-apoptotic effect, with a significantly enriched subG1 population and concomitant cleavage of caspase 3. This effect is surprising, as previous studies of miR-17∼92 have shown direct regulation of ‘pro-apoptotic' molecules Bim and Pten in normal lymphopoiesis,^14^ MYC-driven lymphomas^18, 20^ and immunodeficiency or lymphoproliferative states.^19^ It is interesting to note that while this effect, in MYC-driven lymphoma at least, is primarily mediated by miR-19 family members (miR-19a/b), we have identified principally a miR-17 family- (miR-17, miR-20a/b, miR-106a/b and miR-93) and miR-18 family(miR-18a/b)-driven effect in BCR-ABL-positive ALL on BCL2, indicating differences in pro- and anti-apoptotic functions of miR-17∼92 between the various cellular contexts.

In keeping with the phenotype described for the TonB model, an unbiased global proteomic approach identified several apoptosis-related proteins as miR-17∼19b targets. As targeting the anti-apoptotic pathway offered a logical therapeutic approach, we chose to focus our validation studies on the well-characterized anti-apoptotic protein BCL2. BCL2, an inhibitor of the mitochondrial apoptotic pathway, is the founding member of a large family of BH-domain containing proteins with pro- and anti-apoptotic activities upstream of caspase-activation.^34, 35^ Notably, *Bcl2* induction has previously been shown to be important for prevention of apoptosis in BCR-ABL expressing BaF3 cells.^36^ Our data suggest that downregulation of miR-17∼92 may be an important mediator of this effect. This may also explain why overexpression of the cluster has little phenotypic effect in BCR-ABL-negative ALL cells, where Bcl2 expression may be less critical.

Small molecule BH3-domain mimetica, notably ABT-737^37^ and its orally bioavailable analogue ABT-263 (Navitoclax)^38^ have been designed to inhibit BCL2.^39^ BCL2 inhibition has demonstrated efficacy in pre-clinical and early phase clinical trials in small-cell-lung cancer,^40^ myeloma^41^ and high-risk B cell non-Hodgkin lymphoma.^42^ Furthermore, dramatic responses have been seen in chronic lymphocytic leukaemia,^27^ where development of ABT-263 has produced a potent agent, ABT-199, which avoids the problem of thrombocytopenia seen with earlier agents.^43^ We investigated the efficacy of the first high-affinity BCL2 inhibitor, ABT-737, and have demonstrated a BCR-ABL specific effect in ALL *in vitro*. Furthermore, ABT-737 appeared to be more potent than imatinib in this setting, suggesting that BCL2 inhibition has the potential to improve on current therapeutic regimens. In order to pre-clinically investigate the effect of potential therapies in ALL in a supportive microenvironment, we have developed a real-time murine xenograft model of patient-derived BCR-ABL-positive ALL.^24^ Six weeks of treatment with ABT-737 led to a significantly slower rate of leukaemic progression and associated prolonged survival compared with vehicle treated mice. The increase in dissemination of luciferase-expressing tumour cells following cessation of ABT-737 treatment suggests that even better outcomes could be achieved with on-going treatment. Furthermore, there was no effect on the heart in ABT-737 treated mice (data not shown) or on BCL2 expression in cardiomyocytes *in vitro* (Supplementary Figure 7). These results demonstrate both the *in vivo* efficacy of BCL2 inhibition in BCR-ABL-positive ALL, as well as the potential for our approach in pre-clinical disease modelling.

Our data indicate a selective advantage for low miR-17∼92 expression in primary BCR-ABL-positive ALL cells. Since these miRNAs regulate expression of BCL2 and other apoptosis regulators, the data demonstrate the crucial balance between proliferative signals and apoptosis in BCR-ABL-positive ALL. Our results demonstrate that BCR-ABL expression may result not only in a proliferative state, but also in one which is primed for apoptosis and therefore reliant on upregulation of BCL2. This makes therapeutic targeting of this key anti-apoptotic molecule a logical and attractive option and may provide a therapeutic window relative to normal tissues. Inhibition of BCL2 and BCR-ABL in BCR-ABL-positive ALL cells resulted in different kinetics of cell death, turn-over of BCL2 protein and induction of miR-17∼92 expression. We found that ABT-737 treatment led to a decrease in BCL2 protein levels and an increase in miR-17∼92 miRNA expression in a BCR-ABL-dependent manner, suggesting that BCL2 inhibition results in the perturbation of a complex signalling network. This apparent positive feedback loop may act to augment the effects of pharmacological BCL2 inhibition.

In summary, we have sought to identify potential therapeutic targets in BCR-ABL-positive ALL by validating targets of a miRNA cluster showing downregulated expression and important survival function in this disease. Having validated the anti-apoptotic protein BCL2 as a miR-17∼92 target, we have demonstrated the direct control of *BCL2* expression by miR∼17 and miR∼18 family members by the canonical RNAi effector protein AGO2. Finally, we have demonstrated pre-clinical efficacy of the BCL2 inhibitor, ABT-737, both in an *in vitro* setting and using an *in vivo* model of human BCR-ABL-positive ALL. We suggest that BCL2 inhibition should be considered for early phase clinical testing in BCR-ABL-positive ALL as a new strategy either to improve current standard curative treatments or long-term disease control.

## Supplementary Material

Supplementary Figures and Materials and Methods

Supplementary Table 1A

Supplementary Table 1B
