# Supplementary material for: Differential expression of miR-17∼92 identifies BCL2 as a therapeutic target in BCR-ABL-positive B-lineage acute lymphoblastic leukemia
Source: Leukemia. 2013 Dec 20;28(3):554–65. doi: 10.1038/leu.2013.361 (PMC3948162; doi:10.1038/leu.2013.361)
Supplement: Supplementary Figures and Materials and Methods [file leu2013361x1.doc]

Supplemental Figures

**Figure S1: Differential expression of miR-17~92 in TonB cells after induction of BCR-ABL expression.** Levels of miR-17, miR-18a, and miR-19a were quantified by miR-qRT-PCR. The graph shows expression levels in TonB cells expressing BCR-ABL relative to Ton B cells supplemented with IL-3.


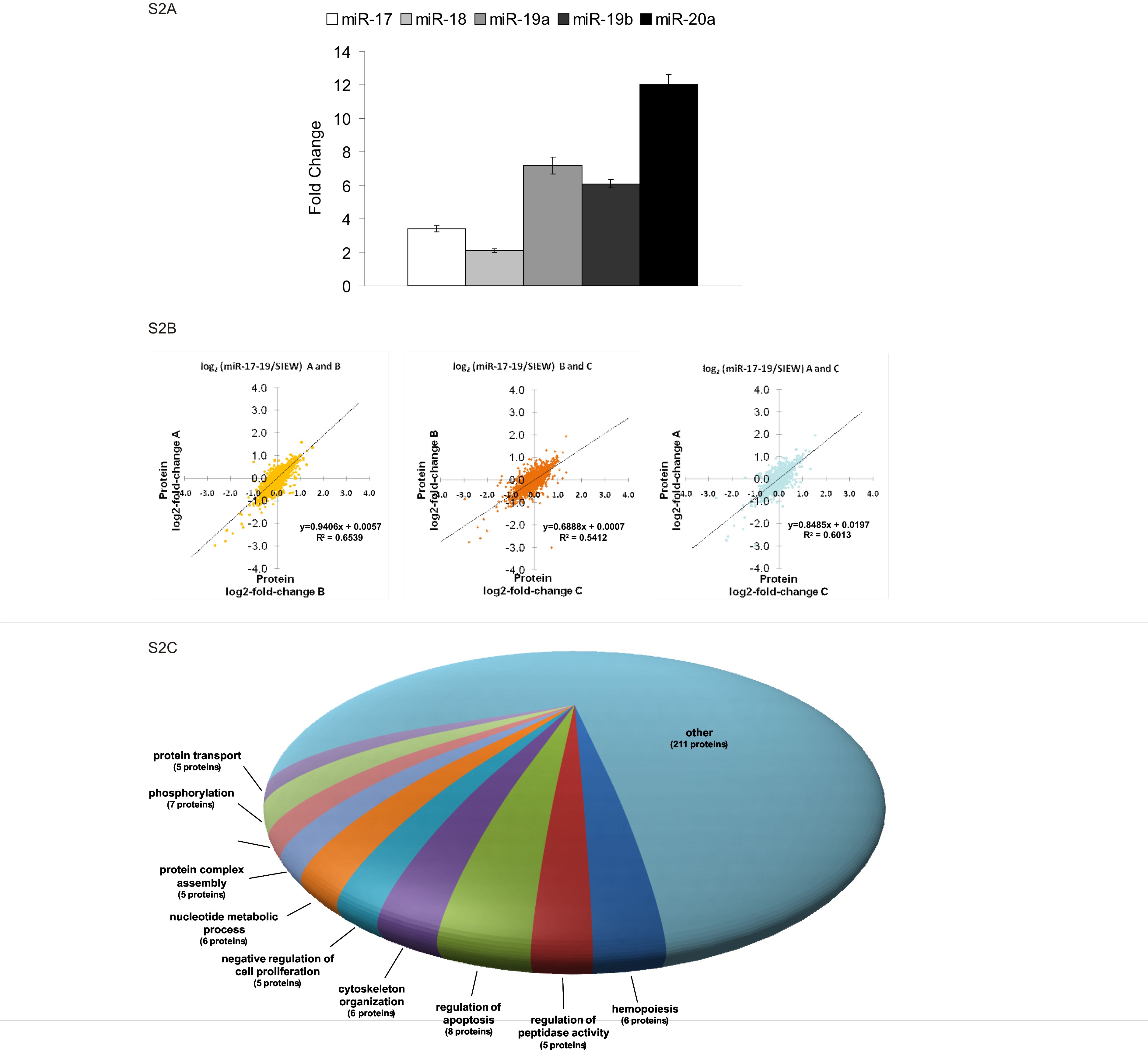


Figure S2: SILAC/LC-MS analysis. (A) Levels of miR-17, miR-18a, miR-19a, miR-19b and miR-20a were quantified by miR-qRT-PCR. The graph shows expression levels in miR-17~19b transduced TonB cells relative to control vector (SIEW) transduced cells (B) Correlation of regulation of protein expression by miR-17~19b compared to control miRNA between the three labelling conditions. **The correlation of protein quantification between two experiments each A and B (left), B and C (middle) and A and C (right) is shown**. (C) Gene ontology analysis: selected gene ontologies showing over-representation of at least 5 proteins per concurrent annotation are shown in detail.

Figure S3: Predicted miR-17~19b binding sites in murine and human *BCL2*. (A) Putative miR-17/20a and miR-18a seed sequences in the 5′UTR or cds of the mouse Bcl2 gene (NM_177410). Upper lane: miR-17/20a or miR-18a consensus; lower lane: corresponding 5′UTR or CDS seed sequence. The folding energy and the position are indicated.

(B) Putative miR-18a and miR-17/20a seed sequences in the CDS of the human BCL2 gene (M13994). Upper lane: miR-18a or miR-17/20a consensus; lower lane: corresponding CDS seed sequence. The folding energy and the position are indicated.

Figure S4: Reduction of BCL2 expression in BCR-ABL positive and –negative ALL cell lines. (A) Western blots depicting protein levels of BCL2 after lentiviral transduction of BCR-ABL positive cell lines Tom-1, SupB15, or BV173 with miR-17~19b or control vector (SIEW). (B) Expression levels of *BCL2* mRNA in BCR-ABL positive cell lines Tom-1, SupB15, or BV173 (left) and BCR-ABL negative cell lines Nalm-6, REH, or 697 (right) after transduction with anti-BCL2 shRNA or ctrl-shRNA, respectively.

**Figure S5: Comparison of ABT-737 and imatinib inhibition in cell lines and primary material.** (A) Western blot depicting protein levels of BCL2 in BCR-ABL positive SupB15 cells after treatment with Imatinib (1 µM) or ABT-737 (0.1 µM) at the indicated time points. (B) The corresponding levels of miR-17, miR-18a, and miR-19 following ABT-737 treatment were determined by miR-qRT-PCR. Graphs show fold change compared to DMSO controls.


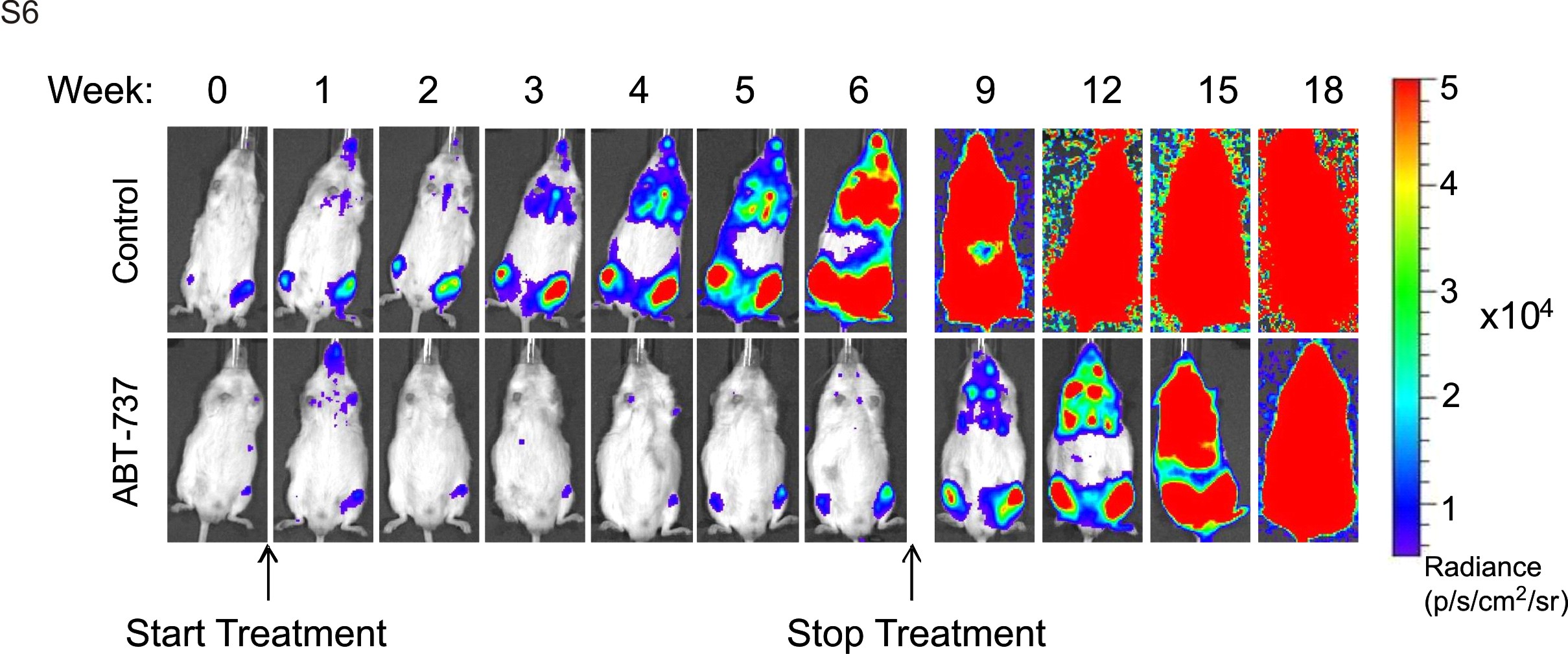


Figure S6: ABT-737 impairs expansion of human primary BCR-ABL+ ALL cells *in vivo*. NSG mice were transplanted with luciferase expressing L4951 blasts and treated with ABT-737 (50 mg/kg/day) or vehicle control for 6 weeks following successful engraftment. Images are representative mice from ABT-737 treated and control groups showing expansion of luciferase expressing transduced blasts over time. This figure is corresponds to Figure 8B but with identical scale for the entire observation period.

Figure S7: ABT-737 has no effects on Bcl2 expression and survival in cardiomyocytes. Western blot depicting protein levels of Bcl2 and cleavage of caspase 3 in neonatal rat cardiomyocytes after treatment with ABT-737 (0.1 µM) after 72h.

Supplemental Methods

Cell Culture

Ton B cells (murine IL-3 dependent pro-B cell line) were cultured in RPMI 1640 supplemented with 10% FCS and 10% WEHI-3B conditioned media as a source of murine IL-3. Expression of BCR-ABL was induced by addition of doxycycline (1µg/ml). 293 and NIH 3T3 cells were cultured in DMEM supplemented with 10% FCS. Lymphoid BCR-ABL positive cell lines BV173, Tom-1, and SupB15, lymphoid BCR-ABL negative cell lines Nalm-6, 697, and REH and primograft L4951 cells were cultured in RPMI 1640 with 10% FCS.

SILAC, LC-MS analysis and data processing

For SILAC analysis, TonB cells were cultured for 18 days in SILAC-DMEM (Silantes, Munich, Germany) supplemented with murine IL-3 (25 ng/ml), 10% dialyzed FCS and either isotopically labelled Lysine (^13^C_6_-^15^N_2_-Lys) and Arginine (^13^C_6_-^15^N_4_-Arg) (heavy state), Lysine (^2^H_4_-Lys) and Arginine (^13^C_6_-Arg) (medium state) or natural Lysine and Arginine (light state). The rate of isotype-specific incorporation of amino acids was determined over time. When labelling exceeded 95%, 1x10^6^ cells were lentivirally transduced with S-miR-17~19b-IEW, S-miR-20a-IEW, or control vector (SIEW) for each labelling condition, facilitating a triplicate analysis with full label switching. After 4 days, the number of EGFP positive cells was analysed by FACS analysis (FACS-Calibur, Becton-Dickinson, Heidelberg, Germany) and the transduction rate was ~99%. Cells were harvested 6 days after transduction by centrifugation and washed three times with PBS. Cells were solubilized in 700 µL of ice cold lysis buffer (8 M urea, 4% CHAPS, 30 mM Tris, pH 8.0, protease inhibitor complete without EDTA (Roche Diagnostics) and sonicated on ice for 10 cycles, 2 seconds each at 10% energy. Cell debris was spun down at 16,000 x g and 4°C for 30 min. Protein content was determined using the Bradford method with bovine serum albumin as the standard. Aliquots of protein extracts were snap-frozen in liquid nitrogen and stored at -80°C until further use. Three biological replicates were prepared with all three labelling states, light, medium and heavy, included. A, S-miR-17~19b-IEW: light, SIEW: heavy. B. S-miR-17~19b-IEW: medium, SIEW: light. C. S-miR-17~19b-IEW: heavy, SIEW medium 33 μg protein was collected from each sample to give a final amount of 99 μg protein, which was subjected to SDS PAGE. Proteins were incubated for 5 min with Laemmli sample buffer at 95°C, alkylated by addition of acrylamide up to a concentration of 2% and incubated at RT for 30 min. SDS PAGE was performed on 12% gels in a mini protean cell (Biorad). After electrophoresis, proteins were stained with Coomassie Brilliant Blue for 15 min and background staining was reduced with water. Lanes were sliced into 12 bands and reduced to 1 mm³ gel pieces. These were destained twice with 200 µL 50% acetonitrile (ACN), 50 mM ammonium bicarbonate (ABC) at 37°C for 30 min and then dehydrated with 100% ACN. Solvent was removed in a vacuum centrifuge and 100 µL 6 ng/µL sequencing grade Trypsin (Promega) in 10% ACN, 40 mM ABC was added. Gels were rehydrated in Trypsin solution for 1 hour on ice and then covered with 10% ACN, 40 mM ABC. Digestion was performed over night at 37°C and was stopped by adding 100 µL of 50% ACN, 0,1% trifluoroacetic acid (TFA). After incubation at 37°C for 1 hour, the solution was transferred into a fresh sample vial. This step was repeated twice and extracts were combined and dried in a vacuum centrifuge. Dried peptide extracts were re-dissolved in 55 µL 2% ACN, 0.1% TFA with shaking at 800 rpm for 20 min. After centrifugation at 20,000 x g aliquots of 12.5 µL each were stored at -20°C.

Peptide samples were separated with a nano-flow ultra-high pressure liquid chromatography system (RSLC, Thermo Scientific) equipped with a trapping column (3 µm C18 particle, 2 cm length, 75 µm ID, Acclaim PepMap, Thermo Scientific). Peptide mixtures were injected, enriched and desalted on the trapping column at a flow rate of 6 µL/min with 0.1% TFA for 5 min. The trapping column was switched online with the separating column of 50 cm length (2 µm C18 particle, 75 µm ID, Acclaim PepMap, Thermo Scientific) and peptides were eluted with a multi-step binary gradient: linear gradient of buffer B (80% ACN, 0.1% formic acid) in buffer A (0.1% formic acid) from 4% to 25% in 115 min, 25% to 50% in 25 min, 50% to 90% in 5 min and 10 min at 90% B. The column was reconditioned to 4% B in 30 min. The Flow rate was 250 nl/min and the column temperature was set to 45°C. The RSLC system was coupled online to a Nano Spray Flex Ion Source II (Thermo Scientific) of an LTQ-Orbitrap Velos mass spectrometer. Metal-coated fused-silica emitters (SilicaTip, 10 µm i.d., New Objectives) and a voltage of 1.2 kV were used for the electrospray. Overview scans were acquired at a resolution of 60k in a mass range of m/z 300-1600 in the orbitrap analyzer and stored in profile mode. The top 10 most intensive ions of charges two or three and a minimum intensity of 2000 counts were selected for collision induced dissociation CID fragmentation with a normalized collision energy of 38.0, an activation time of 10 ms and an activation Q of 0.250 in the LTQ. Fragment ion mass spectra were recorded in the LTQ at normal scan rate and stored as centroid m/z value and intensity pairs. Active exclusion was activated so that ions fragmented once were excluded from further fragmentation for 70 s within a mass window of 10 ppm of the specific m/z value.

Raw data were processed with the MaxQuant proteomics software suite version 1.1.1.36 for identification and quantification of proteins as described. Peptides and proteins were identified with the implemented Andromeda search engine version 1.1.0.36 and the human entries of the IPI protein data base (v. 3.73) at a false discovery rate of 1% at both protein and peptide level. Combined protein ratios were calculated for both biological replicates. A relative change of at least 40% was set as the threshold for significant changes of protein intensities and at least two peptides were required to be quantified in every biological replicate for each protein.

**Construction of lentiviral vectors**

The lentiviral vector S-miR-17~19b-IEW containing the miR-17~19b-1 polycistron, a variant of the miR-17~92 polycistron selected for efficient transgenic miRNA expression which lacks the 3´-located miR-92, has been described previously . shRNAs corresponding to position 345 to 363 of the murine Bcl2 gene (Gene bank accession no. NM_177410), lentiviral transgene plasmids pdc-SR, and shRNA controls were cloned as described . The numbering of the first nucleotide of the shRNA refers to the ATG start codon. Lentiviral constructs encompassing the shRNAs encode RFP (red fluorescent protein) as a reporter gene. Self-complementary DNA oligonucleotides encompassing the sequence of the miRNAs miR-17, miR-18a, and miR-20a, as well as an irrelevant sequence (ant-ctrl) were cloned as described . The preparation of recombinant lentiviral supernatants and lentiviral transductions were performed as described earlier .

**miRNA target verification and luciferase activity assay**

Two DNA oligonucleotides corresponding to predicted targets of miR-17~19b of the sequence of the murine *Bcl2* mRNA (NM_177410) and two oligonucleotides corresponding to predicted targets of miR-17~19b of the sequence of the human *BCL2* mRNA (M13995.1) were chemically synthesized, including overhang sequences from a 5’-XhoI- and a 3’ NotI-restriction site:

miR-17-FP (murine): 5’-TCGAGGAAGATCATGCCGTCCTTAGAAAATACAGTAAGTTCTTTGCACAGGAATTTTGTTTGC-3’

miR-17-RP (murine): 5’-

GGCCGCAAACAAAATTCCTGTGCAAAGAACTTACTGTATTTTCTAAGGACGGCATGATCTTCC-3’

miR-18a-FP (murine): 5’-

TCGAGCCCGCAGCGCACCACACACAGTGCGCGGGCTGCTCCTTGGGCACCCGCGGCCCC GCG-3’

miR-18a-RP (murine): 5’-

GGCCGCGGGGCCGCGGGTGCCCAAGGAGCAGCCCGCGCACTGTGTGTGGTGCGCTGCGGGC-3’

Annealed oligonucleotides were directionally cloned into the XhoI/NotI-digested psiCHECK2 plasmid (Promega, Heidelberg). For each plasmid, the correct sequence and insertion was confirmed by DNA sequencing. Mutant derivatives with deletions in the seed sequence of the miR-17~19b target sites in the murine *Bcl2* mRNA was carried out using the Quick-change mutagenesis kit (Stratagene, Heidelberg, Germany) in which the corresponding wildtype mRNA was used as a template.

NIH3T3 cells stably expressing miR-17~19b (70% confluence) in 24-well plates were transfected with reporter genes using LipofectamineTM2000 (Invitrogen). Cell extracts were prepared 36h after transfection and the luciferase activity was determined using the Dual-Glo Luciferase Assay System (Promega) and Mithras LB 940 luminometer (Berthold technologies). Each sample was measured in duplicate and three sets per sample were performed.

**RNA isolation, miR-qRT-PCR, and qRT-PCR**

Total RNA from cell lines was prepared using Trizol (Invitrogen). Expression of mature miRNAs was determined by miR-qRT-PCR using miRNA specific looped RT-primers and TaqMan probes as recommended by the manufacturer (Applied Biosystems, Foster City, USA). Normalization was performed using the 2-ΔΔCT method relative to U6snRNA. For qRT-PCR of mRNAs, cDNA synthesis was performed with 1µg of total RNA digested with *DNaseI* and subjected to TaqMan-based (Applied Biosystems) gene expression profiling following the manufacturer’s protocol. Primer/probe assays for miR-17~92 pri-miRNA were used as described earlier and for murine Bcl2 (Mm02528810_s1), murine ß2m (Mm00437762_m1), human Bcl2 (Hs01048947_s1), and human ß2m (Hs99999907_m1) were purchased from Applied Biosystems. ß2m served as an internal control. Real-time PCR was performed using an ABI7500 cycler (Applied Biosystems).

Proliferation assay

Cell proliferation was analyzed by the Trypan-blue dye exclusion assay. TonB cells were cultured in 24-well plates at 2x10^4^/ml and human cell lines at 1x10^5^/ml. The number of viable cells was counted after 24-96h by Trypan blue exclusion.

**Apoptosis Assay**

The proportion of apoptotic cells was measured using a modification of the protocol of . In brief, 1 x 10^6^ cells were washed with PBS and fixed in 70% Ethanol. After incubation at 4°C for a minimum of 2 hours, cells were washed with PBS and resuspended in PBS containing 20 µg/ml RNaseA (Qiagen, Hilden, Germany) and 15 µg/ml Propidium iodide (Sigma-Aldrich, Hamburg, Germany). Apoptotic cells were measured by the appearance of a subG1 peak using a FACS Calibur flow cytometer (Beckman Coulter, Heidelberg, Germany).

**Immunhistochemistry**

Cytospins were fixed in cold 4% PFA or ETOH for 10 min. Afterwards slides were briefly air-dried and then washed 3 times each for 5 min in PBS. Cells were permeabilized and blocked for 1h at RT in 1%BSA/10% normal donkey serum/ 0.3M glycine in 0.1%PBS-Tween. The cells were then incubated with the following antibodies overnight 4°C: anti-Bcl2 (ab7973; Stock 0.2mg/ml) 1:25 in PBS and anti-COXIV (ab33985; Stock 1mg/ml) 1:500 in PBS.

The next day cells were washed 3 times with PBS for 5 min. The secondary antibodies were used in a dilution of 1/250 in PBS: anti-rabbit Cy3 (Jackson 711-165-152) and anti-mouse DyLight 488 (Jackson 715-486-151) for 2h at RT. Nuclei were co-stained with DAPI Hoechst 33258 (SIGMA) 1/500 for 5 min. Cells were then washed 3x for 5 min and mounted in Dako fluorescence mounting media.

Images were taken by fluorescence microscopy with Axiovision 4.6 software (Carl Zeiss).

**Immunoblotting**

Whole cell lysates were prepared with lysis buffer (20mM HEPES, pH 7.5, 0.4 M NaCl; 1 mM EDTA, 1 mM EGTA, 1mM DTT) supplemented with mini complete protease inhibitor cocktail tablet (Roche Diagnostics, Mannheim, Germany) and separated by sodium dodecyl sulphate-polyacrylamide gel electrophoresis (SDS-PAGE), transferred to Hybond enhanced chemiluminescence (ECL) nitrocellulose membrane (Amersham Bioscience, Uppsala, Sweden) and membranes were incubated with the following antibodies according to the manufacturer’s protocol: BCL2 (cs2876), SHP-1 (cs3759), GranzymeB (cs4275), β-actin (cs4970), SQSTM1/p62 (cs5114), and Cleaved Caspase-3 (Asp175) (cs 9661) from Cell Signaling Technology; Adseverin (sc-79835), AIF (sc-13116), STAT3 (sc-482), CD43 (M-19) (sc-7054), from Santa Cruz Biotechnology; DNAJB6 from Abcam. Chemiluminescence was used for visualization using the ECL Western blotting detection reagents (Amersham Biosciences) according to the manufacturer. Densitometric analysis of x-ray films was performed using VersaDoc 3000 Imaging system (Bio-Rad) and 1-D analysis software Quantity One Version 4.6.5 (Bio-Rad). The intensity ratio of the protein of interest band to the ß-Actin band (loading control) was calculated to measure changes in protein levels.

**Determination of ABT-737 IC50-values in human cell lines**

For *in vitro* studies, ABT-737 and Imatinib (Selleck Chemicals) were dissolved in DMSO to create a 1 mM stock, and then supplemented to the culture medium at the required concentration. Lymphoid BCR-ABL positive and negative cell lines were plated in 24-well plates at 1.5 X 10^4^ cells/ml in the presence of increasing concentrations of ABT-737 (SelleckBio, Munich, Germany) (0, 0.001, 0.005, 0.001, 0.05, 0.1, 0.5, 1, 5, 10 µM ABT-737). The number of viable cells was determined 48 hours later by PI staining. The number of PI+ cells was plotted against the concentration of ABT-737 and IC_50_-values were calculated using GraphPad Prism software.

**ABT-737 treatment in mouse xenotransplantation studies:**

Primograft material was obtained by serially passaging primary leukemic blasts in NSG mice . Lentiviral transduction of tertiary primograft material was performed as described previously and the transduced material was injected intrafemorally into NSG mice. Mice were imaged using an IVIS Spectrum pre-clinical imaging system (Perkin Elmer) 10 minutes following intraperitoneal injection of 100µl of 30 mg/ml D-Luciferin (Perkin Elmer). Living Image 3.1 Software was used to measure total flux by manually outlining mice in acquired images using region-of-interest tools. ABT-737 (Selleck Chemicals) was dissolved in DMSO (50 mg/ml) and diluted in a vehicle consisting of 65% dextrose water (5% dextrose), 30% propylene glycol and 5% Tween 80. Mice were injected with either vehicle control or ABT-737 (50 mg/kg/day) for a total of 30 days (5 days on, 2 days off). Mice were kept until they exhibited clinical symptoms which necessitated humane killing. Kaplan-Meier curves were plotted and analyzed using GraphPad Prism software, with significance assessed using a Log-rank (Mantel-Cox) test. All work was conducted in accordance with the UK Home Office Project Licence PPL60/3846.

Supplemental References

1. Rehe K, Wilson K, Bomken S, Williamson D, Irving J, den Boer ML*, et al.* Acute B lymphoblastic leukaemia-propagating cells are present at high frequency in diverse lymphoblast populations. *EMBO molecular medicine* 2013 Jan; 5(1): 38-51.

2. Bradford MM. A rapid and sensitive method for the quantitation of microgram quantities of protein utilizing the principle of protein-dye binding. *Analytical biochemistry* 1976 May 7; 72: 248-254.

3. Cox J, Mann M. MaxQuant enables high peptide identification rates, individualized p.p.b.-range mass accuracies and proteome-wide protein quantification. *Nature biotechnology* 2008 Dec; 26(12): 1367-1372.

4. Cox J, Neuhauser N, Michalski A, Scheltema RA, Olsen JV, Mann M. Andromeda: a peptide search engine integrated into the MaxQuant environment. *Journal of proteome research* 2011 Apr 1; 10(4): 1794-1805.

5. Venturini L, Battmer K, Castoldi M, Schultheis B, Hochhaus A, Muckenthaler MU*, et al.* Expression of the miR-17-92 polycistron in chronic myeloid leukemia (CML) CD34+ cells. *Blood* 2007 May 15; 109(10): 4399-4405.

6. Scherr M, Battmer K, Ganser A, Eder M. Modulation of gene expression by lentiviral-mediated delivery of small interfering RNA. *Cell Cycle* 2003 May-Jun; 2(3): 251-257.

7. Scherr M, Venturini L, Battmer K, Schaller-Schoenitz M, Schaefer D, Dallmann I*, et al.* Lentivirus-mediated antagomir expression for specific inhibition of miRNA function. *Nucleic acids research* 2007; 35(22): e149.

8. Nicoletti I, Migliorati G, Pagliacci MC, Grignani F, Riccardi C. A rapid and simple method for measuring thymocyte apoptosis by propidium iodide staining and flow cytometry. *Journal of immunological methods* 1991 Jun 3; 139(2): 271-279.

9. Bomken S, Buechler L, Rehe K, Ponthan F, Elder A, Blair H*, et al.* Lentiviral marking of patient-derived acute lymphoblastic leukaemic cells allows in vivo tracking of disease progression. *Leukemia* 2013 Mar; 27(3): 718-721.
