# Supplementary material for: Differential expression of miR-17∼92 identifies BCL2 as a therapeutic target in BCR-ABL-positive B-lineage acute lymphoblastic leukemia
Source: Leukemia. 2013 Dec 20;28(3):554–65. doi: 10.1038/leu.2013.361 (PMC3948162; doi:10.1038/leu.2013.361)
Supplement: Supplementary Table Legends [file leu2013361x4.doc]

Supplementary table 1A: Proteins regulated by miR-17~19b and miR20a. Proteins were identified using SILAC after ectopic overexpression of the corresponding miRNAs.

Supplementary table 1B: Functional classification of proteins regulated by miR-17~19b and miR20a. **53 low abundance proteins were categorized into functional groups using** GeneCoDis 2.0 using Gene Ontology and KEGG-based gene enrichment analysis.
